# Associations between vaping and daily cigarette consumption among individuals with psychological distress

**DOI:** 10.18332/tpc/189769

**Published:** 2024-06-20

**Authors:** David Estey, Geoffrey F. Wayne, Amanda Sharp, Katie E. Holmes, Rujuta Takalkar, Ana M. Progovac, Benjamin Lê Cook

**Affiliations:** 1 Cambridge Health Alliance, Cambridge, Massachusetts, United States; 2 Department of Psychiatry, Harvard Medical School, Cambridge, Massachusetts, United States

**Keywords:** smoking, vaping, psychological distress, tobacco-related disparities

## Abstract

**INTRODUCTION:**

Individuals with behavioral health conditions smoke at significantly higher rates and have been resistant to existing smoking cessation efforts. A clearer understanding of associations between vaping and daily cigarette consumption in this vulnerable population is warranted.

**METHODS:**

We analyzed data from the 2014–2018 National Health Interview Survey (NHIS) to examine whether vaping was associated with differences in number of cigarettes smoked per day (CPD) among adults who smoke daily and have varying levels of psychological distress.

**RESULTS:**

After adjustment for sociodemographic covariates, individuals who vaped every day smoked on average 1.48 fewer cigarettes per day than individuals who never vaped (p<0.01), while individuals who vaped some days and individuals who ever but no longer vaped smoked 0.77 and 1.48 more CPD, respectively, than individuals who never vaped. Differences between those who vaped every day and those who never vaped were even greater among those with moderate psychological distress (-2.21 CPD, p<0.01).

**CONCLUSIONS:**

Our findings suggest that use of vaping devices may be associated with lower daily cigarette use among individuals with psychological distress, potentially supporting smoking harm reduction efforts.

## INTRODUCTION

Patterns of tobacco use are shifting rapidly in the US and worldwide, with the growing availability of new products, including e-cigarettes (here referred to as vapes)^1,2^ alongside widespread dual- or poly-cigarette tobacco use^3^. While exclusive cigarette smoking remains most common in the US, representing just over 50% of all tobacco use, both exclusive and dual/poly cigarette use rates are declining among adults^4^. By contrast, the use of e-cigarettes, or vaping, is increasing, particularly among younger adults^4^. In 2014–2015, most individuals who vaped (>80%) also used some form of tobacco, but they are now equally likely to vape exclusively^4^.

Debates about the appropriate public health response to the rise in vaping prevalence have centered on the potential health risks of vaping among individuals who smoke (due to regular dual use of vapes and cigarettes) and individuals who do not smoke (through exposure to nicotine and consequent addiction among youth, as well as potential relapse among individuals who have quit smoking)^5,6^. The rise in vaping prevalence also has potential benefits for smoking reduction. Among individuals who currently smoke, vaping is associated with cigarette use reductions and quit attempts, as well as an increased probability of sustained smoking cessation^7^. Ten percent of individuals who smoke also vape^8^, and among those that both smoke and vape, 71% report vaping to help them quit smoking^9^.

Smoking cessation provides the greatest health benefits, but reducing use [e.g. daily use, cigarettes per day (CPD)] can reduce exposure to harmful or potentially harmful constituents and biomarkers of exposure linked to smoking-related disease^10^. Although evidence remains limited, these intermediary outcomes may mitigate the risks of heart disease, lung cancer, and chronic obstructive pulmonary disease (three fatal conditions linked to smoking), as well as overall mortality risks^11^. Reductions in cigarette use have also been shown to increase the probability of making a quit attempt^12^. The effects of reductions in cigarette use are moderated by the scale of change^11^. Thus, while measures of toxicant exposure are reduced among individuals who use both cigarettes and vapes compared to those who smoke exclusively, cigarette consumption remains the primary driver of toxicant exposure, with high exposures among those who continue to smoke daily^13^. Dual product use may also offset some of the potential benefits of reductions in cigarette use^14^. For example, vaping supports nicotine intake and overall nicotine dependence, even with a reduction in smoking, potentially undermining cessation^15^.

Vaping’s potential for harm reduction may be greatest among vulnerable populations of smokers, such as individuals living with mental health challenges or regular non-nicotine substance use that have been resistant to existing smoking cessation efforts. Individuals with behavioral health conditions, including substance use, depression, anxiety, and serious mental illness, are significantly more likely than the general population to smoke cigarettes, leading to higher comorbid physical illnesses and lower life expectancy^16^. They are also more likely to have tried vaping and to currently vape, whether they have never smoked or are currently smoking^17^. Vaping during smoking cessation treatment is common for those with behavioral health conditions, and dual-use of cigarettes and vapes is high among individuals seeking mental health or substance use treatment services, with evidence suggesting vaping can be used successfully as a quitting aid within this population^18^. Given that individuals with behavioral health conditions may be more likely to use vaping devices, and vaping may help these individuals reduce or quit smoking, a clear understanding of whether vaping helps reduce cigarette consumption in this vulnerable population is warranted.

Serious psychological distress (SPD) is a commonly used indicator of non-specific psychological distress predictive of serious mental illness^19^. People with SPD demonstrate characteristics of decreased daily functioning, lower socioeconomic status, higher comorbidities, and healthcare utilization rates similar to those diagnosed with a serious mental illness (e.g. severe major depression, schizophrenia)^20^. SPD is associated with higher rates of smoking and nicotine dependence, as well as lower quit rates and higher rates of relapse after quitting^21^. Cross-sectional studies of US adults report levels of cigarette, vaping, and dual product use among those with SPD more than twice that of those without SPD, with an increased likelihood of both current vaping and daily vaping^22,23^. Less is known about the relationship between vaping and smoking among individuals with moderate levels of psychological distress (MPD), a group whose members may not experience serious mental illness but still endorse higher rates of mental healthcare utilization, impairment, substance use, and other risks compared to those with no/low psychological distress^24^.

In this study, we extend prior findings linking psychological distress to daily cigarette consumption and vaping patterns in two ways: 1) we focus on current daily smokers, examining the associations between frequency of vaping (every day, some days, ever but no longer, never) and quantity of cigarettes smoked (average number of cigarettes smoked per day); and 2) we compare cigarette consumption/vaping by three levels of psychological distress (no/low psychological distress (NPD), moderate psychological distress (MPD), and serious psychological distress (SPD), so that interventions can be better targeted towards individuals with differing levels of mental health. We hypothesized that more frequent vaping would be associated with lower daily cigarette consumption and that this association would be stronger among those with increased psychological distress. Given the substantially higher rates of vaping within younger age cohorts, we also explored the relationships between vaping, cigarette consumption, and psychological distress by age.

## METHODS

### Data

We pooled data from the 2014–2018 National Health Interview Survey, an annual cross-sectional household surveys representative of the US non-institutionalized population that capture health behaviors and health status measures including tobacco use and psychological distress^8^. Data across the 2014–2018 survey years were combined to increase sample sizes to stabilize estimates. We restricted the sample to current daily smokers, determined by the response to the question: ‘Do you currently smoke?’, among adults aged ≥18 years who also endorsed at least one current cigarette smoked per day, drawn from the sample adult file of the NHIS (n=24429).

### Main outcome: current cigarettes smoked per day (CPD)

Our main outcome of interest was current cigarettes smoked per day (CPD), determined by the numerical response to the question: ‘On average, how many cigarettes do you now smoke a day?’. CPD is a commonly used item in measurements of cigarette dependence (e.g. Fagerström test for nicotine dependence (FTND), Cigarette Dependence Scale, Penn State Cigarette Dependence Index), with higher CPD associated with higher dependence.

### First primary exposure: level of psychological distress

Our first primary exposure of interest was the level of psychological distress as measured by the Kessler 6-Item Psychological Distress Scale (K-6), [no/low psychological distress (NPD: K-6 <5), moderate psychological distress (MPD: K-6 >5 and <13), and serious psychological distress (SPD: K-6 >13)]^19^. A score ≥13 on the K-6 suggests the presence of serious mental illness defined as meeting DSM-IV criteria for a mental health disorder in the past 12 months and a Global Assessment of Functioning score of <60^19^. A ‘moderate’ psychological distress score (K-6 score = 5–12) is indicative of mental distress that may not be linked to a clinical diagnosis (i.e. sub-threshold), but is still accompanied by impairment across a range of functional domains (e.g. employment, household, relationships)^24^.

### Second primary exposure: vaping frequency

Our second primary exposure of interest was vaping frequency, operationalized as individuals who vape every day (every-day vapers), individuals who vape some days (some-days vapers), individuals who vaped at least once but no longer vape (ever but no longer vapers), and individuals who never vaped (never vapers), determined by the two questions: ‘Have you ever used an e-cigarette, even 1 time?’ and ‘Do you now use e-cigarettes every day, some days, or not at all?’^25^.

### Covariates

Covariates were added to regression models that have been shown to be independently associated with smoking, including age (<25, 25–44, ≥45 years of age), sex (male, female), sexual orientation (lesbian or gay, straight, bisexual, other), race (White, Black, American Indian/Alaskan Native, Asian, Multiple Race), ethnicity (Hispanic/non-Hispanic), marital status (married, separate/divorced, widowed, living with partner, never married), and family combined income ($) (0–49999, 50000–99999, ≥100000). Missingness for sociodemographic covariates ranged from 1.4% to 6.1% across covariates. In all, 1963 cases with data missing from at least one covariate were omitted from analyses, resulting in a final sample size of n=22466.

### Analytical approach

First, we compared levels of psychological distress and sociodemographic covariates by frequency of vaping (every day, some days, ever but no longer, and never) using chi-squared tests to determine significance of omnibus differences across all categories.

Second, we estimated multivariable linear regression models of cigarettes smoked per day, conditional on the frequency of vaping (categorized as every-day, some-days, ever but no longer, never), psychological distress (categorized as NPD, MPD, and SPD), the interaction of vaping and psychological distress (these variables were demeaned, or centered so that main effect coefficients could be directly interpreted^26^), and other covariates described above. The coefficient on the term representing the interaction of vaping frequency and psychological distress provides information on whether the associations between vaping frequency and cigarettes per day was greater among individuals with psychological distress (moderate and serious; reference: none/low).

Analyses were conducted using SAS (ver. 9.4) SURVEY procedures to account for NHIS survey weights so that estimates are representative of the US non-institutionalized adult population.

## RESULTS

Approximately half of the individuals who reported currently smoking also reported ever vaping (11291 individuals reporting ever but no longer, some days or every day vaping out of 22466 individuals currently smoking) (Table 1). Rates of moderate and serious psychological distress were lower for never vapers, compared to ever but no longer vapers, some-days and every-day vapers (p<0.01) (Table 1). Never vapers also significantly differed from ever but no longer, some-days and every-day vapers across demographic categories. Never vapers were older, less likely to report lesbian or gay sexual orientation, more likely to self-identify as Hispanic ethnicity and Black race, less likely to have never married, and had lower income compared to the other vaping groups (Table 1).

**Table 1:** Levels of psychological distress and sociodemographic characteristics by vaping frequency among current smokers (N=22466)

		*Frequency of vaping*				
	*Total %*	*Every day (N=644) %*	*Some days (N=2046) %*	*Ever but no longer vape (N=8601) %*	*Never vaped (N=11175) %*	*p*
**Psychological distress**						<0.01
No/low	65.32	58.36	59.41	60.16	70.88	
Moderate	25.76	30.98	29.60	29.44	21.84	
Serious	8.93	10.66	10.99	10.40	7.28	
**Age (years)**						<0.01
<25	7.76	11.85	12.00	9.68	5.22	
25–44	38.68	43.45	43.29	45.53	32.14	
≥45	53.56	44.70	44.71	44.79	62.63	
**Sex assigned at birth**						0.05
Male	52.26	56.03	53.09	51.11	52.79	
Female	47.74	43.97	46.91	48.89	47.21	
**Sexual orientation**						<0.01
Lesbian or gay	2.60	2.50	3.20	3.26	1.96	
Straight, that is, not lesbian or gay	94.55	93.93	92.89	93.59	95.65	
Bisexual	1.67	2.67	3.11	1.96	1.11	
Other	1.19	0.89	0.80	1.19	1.28	
**Ethnicity**						<0.01
Hispanic/Spanish	9.34	5.80	7.56	7.08	11.66	
Non-Hispanic/Spanish	90.66	94.20	92.44	92.92	88.34	
**Race**						<0.01
White only	79.68	85.99	85.35	83.65	75.13	
Black/African American only	13.58	6.35	8.09	9.50	18.22	
American Indian/Alaskan Native only	1.30	1.66	0.77	1.12	1.52	
Asian only	2.76	2.62	2.54	2.49	3.02	
Multiple race	2.68	3.38	3.24	3.23	2.11	
**Marital status**						<0.01
Married	30.79	30.13	28.24	30.83	31.26	
Separated or divorced	24.95	22.09	23.90	23.53	26.43	
Widowed	6.70	5.64	6.20	4.71	8.42	
Living with partner	10.96	13.09	13.39	12.68	9.02	
Never married	26.61	29.04	28.28	28.24	24.87	
**Family combined income ($)**						<0.01
0–49999	60.67	57.21	58.41	58.35	63.11	
50000–99999	24.38	26.52	26.77	25.39	23.01	
≥100000	14.96	16.27	14.82	16.26	13.87	

Percentages are column %. Data obtained from pooled 2014–2018 National Health Interview Surveys. Percentages reported are weighted to account for NHIS survey weights. Psychological distress measured using the Kessler 6 scale. P-values represent tests of significance of omnibus chi-squared tests, assessing differences between observed and expected values in cross-tabulation comparisons.

### CPD by vaping frequency (unadjusted)

Every-day vapers smoked fewer cigarettes per day than other categories of vapers across all categories of psychological distress (Supplementary file Table S1).

### CPD by psychological distress (unadjusted)

Adults who smoked with no psychological distress reported fewer cigarettes per day than individuals with moderate psychological distress, who in turn reported fewer cigarettes per day than individuals with serious psychological distress (Supplementary file Table S1).

### CPD by psychological distress and vaping frequency (unadjusted)

**Figure 1:**
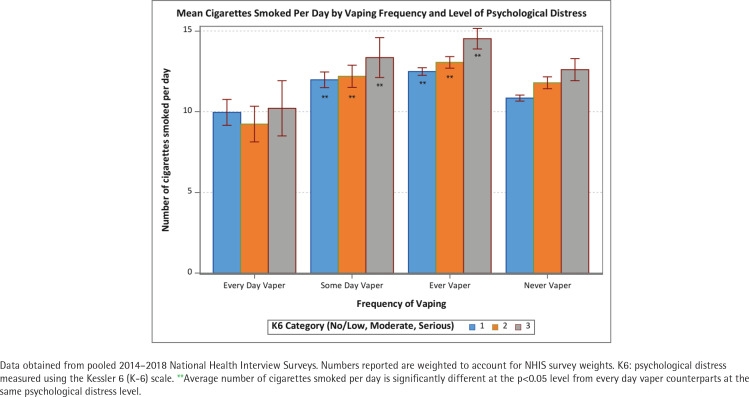
Mean number of cigarettes smoked per day among current smokers by vaping frequency and level of psychological distress, all ages (N=22466)

**Figure 2:**
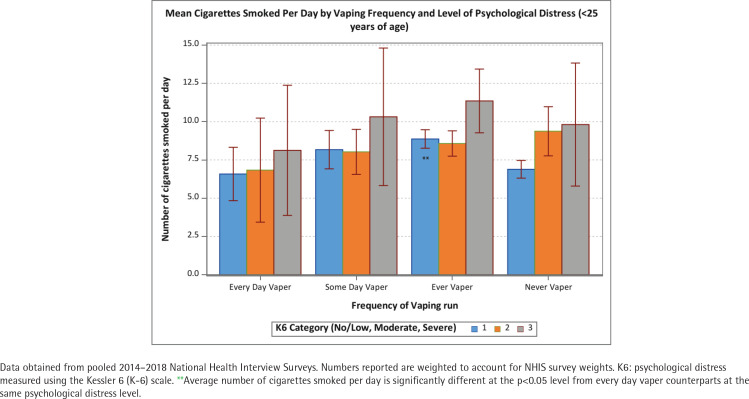
Mean number of cigarettes smoked per day among current smokers by vaping frequency and level of psychological distress (<25 years of age)

**Figure 3:**
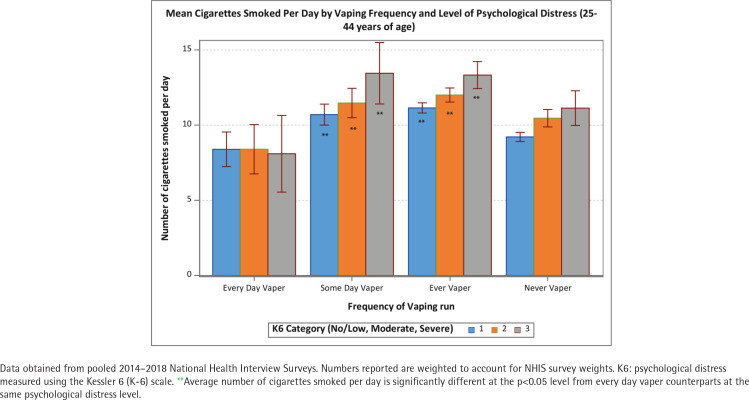
Mean number of cigarettes smoked per day among current smokers by vaping frequency and level of psychological distress (25–44 years of age)

**Figure 4:**
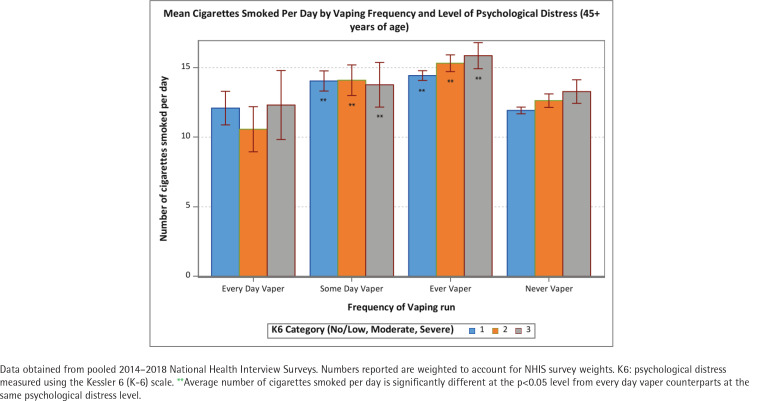
Mean number of cigarettes smoked per day among current smokers by vaping frequency and level of psychological distress (≥45 years of age)

The positive association between psychological distress and cigarettes per day was held among never and former vapers (p<0.05) but not among some-days and every-day vapers (Figure 1). The number of CPD was approximately 10 for all levels of psychological distress among every-day vapers. These patterns were similar across age groups (<25, 25–44, and ≥45 years) (Figures 2–4). Of note is that the gap in CPD between those with no psychological distress and severe psychological distress was much wider among former vapers and never vapers in the <25 years age cohort.

### CPD by vaping frequency (adjusted)

In multivariable linear regression models (Table 2), after adjustment for all above-described covariates, every-day vapers smoked 1.48 fewer CPD than never vapers (p<0.01). Some-days vapers and ever but no longer vapers smoked 0.77 more CPD and 1.48 more CPD, respectively, than never vapers (both differences significant at the p<0.01 level).

**Table 2:** Summary of adjusted multiple linear regression measuring association of cigarettes smoked per day by vaping frequency, levels of psychological distress, and interaction of vaping frequency by psychological distress (N=22466)

	*β*	*SE*	*95% CI*	*p*
**Intercept**	10.62	0.25	10.13–11.19	<0.01
**Vaping frequency**				
Every day	-1.48	0.40	-2.26 – -0.70	<0.01
Some days	0.77	0.24	0.29–1.24	<0.01
Ever but no longer vaping	1.48	0.15	1.18–1.78	<0.01
**Psychological distress**				
Moderate	0.91	0.16	0.59–1.23	<0.01
Serious	1.98	0.26	1.47–2.49	<0.01
**Vaping frequency × Psychological distress**				
Every day × Moderate	-2.21	0.81	-3.79 – -0.63	<0.01
Every day × Serious	-1.74	1.05	-3.80–0.32	0.09
Some days × Moderate	-1.08	0.54	-2.13 – -0.02	<0.05
Some days × Serious	0.05	0.92	-1.76–1.86	0.96
Ever × Moderate	-0.08	0.34	-0.75–0.59	0.81
Ever × Serious	0.03	0.52	-0.99–1.05	0.96

Reference groups are never vaping, none/low psychological distress. SE: standard error. Data obtained from pooled 2014–2018 National Health Interview Surveys. Beta coefficients reported are weighted to account for NHIS survey weights. Psychological distress measured using the Kessler 6 scale. P-values represent tests of significance of estimated regression coefficients in the multiple linear regression model.

### CPD by psychological distress (adjusted)

Those with moderate and severe psychological distress smoked 0.91 more CPD and 1.98 more CPD, respectively, than those with no psychological distress (both differences p<0.01).

### CPD by psychological distress and vaping frequency (adjusted)

Assessing interaction coefficients, the positive association between moderate psychological distress and cigarettes per day was diminished among every-day vapers (-2.21 CPD, p<0.01) and some-days vapers (-1.08 CPD, p<0.05). No other interaction coefficients between vaping frequency and psychological distress were significant.

While not a primary research question, the multivariable linear regression model identifies covariates that are significantly correlated with number of cigarettes smoked per day (Supplementary file Table S2). Adjusting for all other variables, older age was associated with more cigarettes per day. Females smoked 2.2 fewer cigarettes per day than males, and individuals identifying as straight smoked more cigarettes than those identifying as bisexual or ‘Other’ regarding sexual orientation. Whites smoked more cigarettes than all other races aside from those identifying as ‘Multiple races’. Individuals who were never married smoked <1 cigarette fewer than individuals of other marital status. Similarly, individuals with $50000–99999 combined income households smoked <1 fewer cigarettes than those in <$50000 combined income households.

## DISCUSSION

This study builds on prior findings regarding relationships between cigarette smoking, vaping, and psychological distress by focusing on daily cigarette use among individuals with varying frequencies of vaping and varying levels of psychological distress. Consistent with other studies, we found that higher severity of psychological distress was associated with both a higher likelihood of ever vaping and greater number of cigarettes per day. Approximately 50% of all individuals currently smoking had vaped at least once, with this proportion increasing to 60% among those with any psychological distress, indicating that dual use of smoking and vaping was especially common among individuals with moderate or serious psychological distress.

### Vaping and smoking

Those in the US who have tried vaping at least once smoked more CPD, on average, compared to individuals who had never vaped. Individuals who have tried vaping may represent smokers with higher dependence on tobacco/nicotine relative to those who have never vaped or may consist of populations who are at higher risk of smoking more heavily. Individuals who have tried vaping may also represent a subset of adults who smoke and are seeking to reduce or discontinue smoking by transitioning to a tobacco-free source of nicotine. Vaping has been considered an alternative to cigarette smoking for those looking to quit, and smoking cessation or reduced consumption continues to be a primary motivator for initiation or continued vaping among adults who continue to smoke cigarettes^27,28^.

As hypothesized, more frequent vaping was associated with fewer CPD among those who have tried vaping. Individuals who had ever but no longer vaped reported the highest number of CPD across the full sample. Among those who continued to vape, those who vaped daily reported fewer CPD than those who vaped only on some days. These findings support the association of daily (vs non-daily) vaping with improved odds of successfully quitting smoking demonstrated in the extant literature^29^. Alternatively, the finding of fewer CPD among daily vapers could be attributed to newer cohorts of smokers who vape who have always smoked fewer cigarettes. Our age subgroup analyses partially refute this interpretation; however, in-depth longitudinal studies tracking smoking and vaping behaviors are needed to conclusively determine whether vaping leads to reduced smoking.

Most dual users identify harm reduction (whether quitting or reducing the number of cigarettes smoked) as a reason for dual use^9^. The high proportion (41%) of adults currently smoking who reported ever but no longer vaping may reflect curiosity to try among those without intent to quit or a trend of ‘trying-then-discontinuing’ smoking cessation methods, particularly among individuals with high levels of cigarette dependence, who are less likely to quit successfully than those with low cigarette dependence^30^. Those who ever vaped may be at higher risk of developing smoking-related health issues, given higher levels of cigarette consumption when compared to adults who smoke who continued to vape. Some individuals who ‘tried-and-continued’ to vape may also represent smokers who faced fewer barriers to reduced cigarette use or cessation (e.g. low dependence levels, high self-efficacy, no depression or anxiety, supportive social network)^31^.

### Vaping, smoking, and psychological distress

As in previous studies, we identified that individuals with psychological distress smoked more CPD than those with no psychological distress. The higher prevalence of smoking amongst individuals with mental illness is well-established and likely bi-directional (e.g. increased mental health symptoms may lead to increased smoking as a coping strategy, and the onset of smoking may result in poorer physical health/quality of life, leading to symptoms of depression and anxiety). The higher proportion of individuals who tried vaping at least once identified among smoking individuals with moderate or serious psychological distress, compared to those with no psychological distress, is consistent with previous findings in the general population regarding vaping and psychological distress, suggesting there is bi-directionality between mental illness and both types of nicotine delivery under study^22,23^.

Interactions between psychological distress and vaping suggest that the positive association between psychological distress and CPD was diminished among individuals with moderate psychological distress who vaped every day or some days. These findings indicate that current vaping is not only associated with smoking fewer CPD among individuals who currently smoke but that there is an even greater CPD reduction among those with moderate psychological distress. Despite this association, this modest reduction (1–2 cigarettes per day) may not reduce exposure to harmful substances in these individuals and may not reduce their risk of harm from cigarette smoking^10^.

In contrast, vaping frequency did not affect the association between psychological distress and CPD among individuals with serious psychological distress. Thus, our hypothesis that the association between more frequent vaping and fewer CPD would be stronger among individuals with increased psychological distress was partially supported, as this was the case for individuals with moderate but not serious psychological distress. Additional assessment of vaping patterns (frequency of use on days used, e-liquid nicotine concentration, type of device used, triggers for use) could help clarify this partial association.

### Vaping, smoking, and age

Prevalence of ever vaping decreased with age, with 67% of individuals aged <25 who currently smoke reporting vaping at least once versus 59% of those aged 25–44 years and 42% of those aged ≥45 years. Although these findings appear consistent with the higher prevalence of vaping among younger versus older populations, it is important to note that our sample consisted solely of individuals who were current smokers – thereby not capturing non-smoking vapers and limiting the conclusions to be drawn regarding overall vaping patterns by age.

Older individuals who smoked (aged ≥45 years) consumed more CPD and were less likely to have tried vaping compared to younger individuals who smoked. Older individuals who smoked who had ever but no longer vaped represented the group with the highest CPD in our sample, suggesting that this group may be at the highest risk of negative health effects related to cigarette smoking and are less likely to benefit from the potential effects of vaping on daily cigarette consumption (due to their discontinued vaping).

Adult vaping has been generally constant throughout the period of the present analysis (3.3% prevalence in 2014 vs 3.2% in 2018) but rose to 4.5% in 2019 and has maintained that level since, with increases concentrated among those aged 18–24 years (from 7.6% to 11% from 2018 to 2021) and to a less extent among those aged 25–44 years (from 4.3% to 6.5%)^8^. We anticipate that continued increases in vaping, concentrated primarily among younger populations, will further support the substitution of some or all cigarette use among those younger people who smoke, extending the trends found in the present study.

### Vaping and potential harm reduction

Individuals with psychological distress are particularly vulnerable to the harmful effects of smoking, given both greater dependence on nicotine and greater difficulty in quitting^32^. The benefits to increasing smoking cessation rates in this population are clear, and smoking cessation should remain a primary public health goal. Our findings suggest that vaping may indirectly contribute to this goal. By frequently vaping nicotine, individuals with psychological distress may be reducing their reliance on smoking combustible cigarettes for nicotine intake (a common driver of cigarette smoking)^33^. This is similar to the experience of nicotine-replacement therapy (NRT), although traditional NRTs involve other methods of nicotine intake (e.g. transdermal, orally, nasal). Initially, NRTs were not meant to be used while concurrently smoking cigarettes, and those attempting to quit were directed to only engage in NRT use on or after their quit date. In 2013, the Food and Drug Administration (FDA) announced a change in NRT labeling to reflect the feasibility and safety of engaging in dual NRT/ cigarette smoking for a period of time. Referred to as ‘pre-quit NRT use’ by Fucito et al.^34^, this period where NRT and cigarette smoking overlap may allow those who smoke to begin reducing overall cigarette smoking as part of their quit attempt^34^. Should vaping mimic these transitional effects of traditional NRTs, it is possible that individuals who vape frequently may see reductions in CPD during this pre-quit period of dual-use, and may be building up to eventual cessation.

Harm reduction, particularly in the form of cutting back substance use as a step toward eventual cessation, is an alternative to immediate abstinence and has well-documented success with both illicit and licit substance use behavior change^35^. This step-wise approach may lower adverse consequences associated with risky substance use behaviors (including smoking) while maintaining engagement and motivation as a person progresses through the stages of change^36^. Given younger individuals who smoke already smoke fewer CPD and are much more likely to vape and vape every day, we may see the most readiness for change within this population, i.e. to complete switching to vaping, if not eventual cessation of all nicotine products. This is supported by trend-based analysis that shows both reduced cigarette use and higher vaping in younger populations over time^4^. Further study is needed to understand underlying beliefs, motivations, and patterns of poly-tobacco use among younger populations living with psychological distress.

### Limitations

In 2019, the National Center for Health Statistics redesigned the National Health Interview Survey and introduced a new format of annual core questions and rotating core questions that only appear in the survey every few years. The psychological distress scale used in the present study to capture NPD/MPD/SPD was relegated to the rotating core and was unavailable for the 2019 and 2020 surveys. Only limited mental health measures (PHQ-9, GAD-7) were available in these later surveys. For this reason, the present study was necessarily limited to the 2014–2018 period. The age of the dataset is a potential limitation, particularly given the rapid evolution of vaped products with respect to the efficiency of nicotine delivery and ease of operation, although most meaningful (‘fourth generation’) product advances had been established by the end of the study period. As discussed above, we anticipate further adoption of vaped products led primarily by younger populations to extend the trends found in the present study.

For the purposes of this study, data from the National Health Interview Survey only permitted cross-sectional analyses and causality cannot be inferred. Temporal information (i.e. age of onset of vaping, cigarette smoking, psychological distress) and information on motivation leading up to onset (and discontinuation) of vaping, would have provided additional context regarding the role of vaping in daily cigarette consumption. Data on individual vaping behaviors were limited. Given the high interindividual variability in vaping, additional information on specific vaping patterns (frequency/quantity of vaping per day, percentage of nicotine in vaping liquid, type of device used) could further clarify the association between vaping and smoking among individuals with psychological distress.

## CONCLUSIONS

Our findings suggest that use of vaping devices may be associated with lower daily cigarette use among individuals with psychological distress, potentially supporting smoking harm reduction efforts within this population.

## Supplementary Material



## Data Availability

The data supporting this research are available from the authors on reasonable request.
