# Corrigendum: Associations between vaping and daily cigarette consumption among individuals with psychological distress

**DOI:** 10.18332/tpc/193846

**Published:** 2024-11-01

**Authors:** David Estey, Geoffrey F. Wayne, Amanda Sharp, Katie E. Holmes, Rujuta Takalkar, Ana M. Progovac, Benjamin Lê Cook

**Affiliations:** 1 Cambridge Health Alliance, Cambridge, Massachusetts, United States; 2 Department of Psychiatry, Harvard Medical School, Cambridge, Massachusetts, United States

**Keywords:** smoking, vaping, psychological distress, tobacco-related disparities

Corrigendum on:

Associations between vaping and daily cigarette consumption among individuals with psychological distress

By David Estey, Geoffrey F. Wayne, Amanda Sharp, Katie E. Holmes, Rujuta Takalkar, Ana M. Progovac, Benjamin Lê Cook

Tobacco Prevention & Cessation, Volume 10, Issue June, Pages 1-12

Publish date: 20 June 2024

DOI: https://doi.org/10.18332/tpc/189769

The original article authors’ list is now corrected; the last name Wanye is now replaced with its correct spelling as Wayne; the author Katie E. Holmes and superscript for her affiliation are inserted after the author Amanda Sharp; and Ana Progovac is replaced by Ana M. Progovac. The author Katie E. Holmes has completed and submitted the ICMJE Form for Disclosure of Potential Conflicts of Interest and none was reported. All the authors (including K.E. Holmes) report that Katie E. Holmes meets all four criteria for authorship and that the omission of the name during publication was inadvertent. The mentioned changes have also been completed online.

