# Supplementary material for: Associations between vaping and daily cigarette consumption among individuals with psychological distress
Source: Tob Prev Cessat. 2024 Jun 20;10:10.18332/tpc/189769. doi: 10.18332/tpc/189769 (PMC11188481; doi:10.18332/tpc/189769)
Supplement: Supplementary file 1 [file TPC-10-25-s1.pdf]

Supplementary Table S1: Mean number of cigarettes per day smoked by vaping frequency and level of psychological distress (n = 22466)

		Frequency of Vaping			
**Psychological Distress**	**Total**	**Every Day** (n = 644)	**Some Day** (n = 2046)	**Ever but no longer** (n = 8601)	**Never** (n = 11175)
**None/Low** (n = 14699)	11.52 [11.33 – 11.70]	10.43 [9.39 – 11.46]	12.03 [11.4 2– 12.65]	12.43 [12.14 – 12.73]	10.87 [10.64 – 11.11]
**Moderate** (n = 5762)	12.32 [12.02 – 12.63]	9.01 [7.76 – 10.26]	11.74 [10.93 – 12.54]	13.16 [12.70 – 13.62]	11.85 [11.43 – 12.28]
**Serious** (n = 2005)	13.56 [13.05 - 14.07]	10.27 [8.39 – 12.16]	13.65 [12.05 – 15.25]	14.30 [13.60 – 15.00]	12.98 [12.18 – 13.77]
**Total**		9.97 [9.21 – 10.74]	12.12 [11.65 -12.59]	12.84 [12.59 – 13.09]	11.24 [11.04 – 11.45]

Notes: Data obtained from pooled 2014-2018 National Health Interview Surveys. Numbers reported are weighted to account for NHIS survey weights.
Psychological distress measured using the Kessler 6 (K-6) scale.

Supplementary Table S2: Adjusted multiple linear regression measuring association of cigarettes smoked per day by vaping frequency, levels of psychological distress, interaction of vaping frequency by psychological distress, age, and demographic variables (sex assigned at birth, sexual orientation, ethnicity, race, marital status, family income) (n = 22,466)

	**β**	**Std. error**	**95% CI**	***p-value***
**Intercept**	10.62	0.25	10.13 - 11.11	<0.01
**Vaping Frequency**				
Every Day Vaping	-1.48	0.40	-2.26 - -0.70	<0.01
Some Day Vaping	0.77	0.24	0.29 - 1.24	<0.01
Ever But No Longer Vaping	1.48	0.15	1.18 - 1.78	<0.01
**Psychological Distress**				
Moderate. PD	0.91	0.16	0.59 - 1.23	<0.01
Serious PD	1.98	0.26	1.47 - 2.49	<0.01
**Vaping Frequency*Psychological Distress**				
Every Day Vaping*Moderate PD	-2.21	0.81	-3.79 - -0.63	<0.01

Every Day Vaping*Serious PD	-1.74	1.05	-3.80 - 0.32	0.09
Some Day Vaping* Moderate PD	-1.08	0.54	-2.13 - -0.02	<0.05
Some Day Vaping* Serious PD	0.05	0.92	-1.76 - 1.86	0.96
Ever Vaping*Moderate PD	-0.08	0.34	-0.75 - 0.59	0.81
Ever Vaping*Serious PD	0.03	0.52	-0.99 - 1.05	0.96
**Age**				
25-44 years old	2.25	0.24	1.78 - 2.72	<0.01
45+ years old	4.59	0.26	4.08 - 5.10	<0.01
**Sex Assigned at Birth**				
Female	-2.20	0.14	-2.48 - -1.93	<0.01
**Sexual orientation**				
Lesbian or gay	-0.79	0.44	-1.66 - 0.07	0.07
Bisexual	-0.89	0.44	-1.75 - -0.03	<0.05
Other	-1.66	0.53	-2.70 - -0.62	<0.01
**Ethnicity**				
Hispanic/Spanish	-5.12	0.21	-5.52 - -4.71	<0.01
**Race**				
Black/African American only	-3.90	0.19	-4.27 - -3.54	<0.01
American Indian/Alaskan Native only	-2.97	0.49	-3.93 - -2.02	<0.01
Asian only	-4.61	0.33	-5.26 - -3.95	<0.01
Multiple race	-0.48	0.44	-1.35 - 0.39	0.28
**Marital Status**				
Married	0.68	0.19	0.32 - 1.04	<0.01
Separated or divorced	0.70	0.21	0.29 - 1.10	<0.01
Widowed	0.79	0.34	0.12 - 1.46	<0.05
Living with partner	0.67	0.22	0.24 - 1.11	<0.01
**Family Combined Income**				
$50,000 - $99,999	-0.79	0.17	-1.12 - -0.47	<0.01
$100,000 and over	-0.45	0.20	-0.84 - -0.06	<0.05

Notes: Reference groups are never vaping, low psychological distress, <25 years of age, male, straight (that is not lesbian or gay, or bisexual), non-Hispanic/Spanish, White only, never married, and <$50,000.
β: Beta coefficient, Std. error: Standard error, 95% Cl: 95% Confidence Interval, PD: Psychological Distress.
Data obtained from pooled 2014-2018 National Health Interview Surveys.
Beta coefficients reported are weighted to account for NHIS survey weights.
Psychological distress measured using the Kessler-6 scale.
P-values represent tests of significance of individual estimated regression coefficients in the multiple linear regression model.

© 2024 Estey D. et al.
